# Supplementary figures and images for: Marker Assisted Selection of Malic-Consuming Saccharomyces cerevisiae Strains for Winemaking. Efficiency and Limits of a QTL’s Driven Breeding Program
Source: J Fungi (Basel). 2021 Apr 15;7(4):304. doi: 10.3390/jof7040304 (PMC8071496; doi:10.3390/jof7040304)

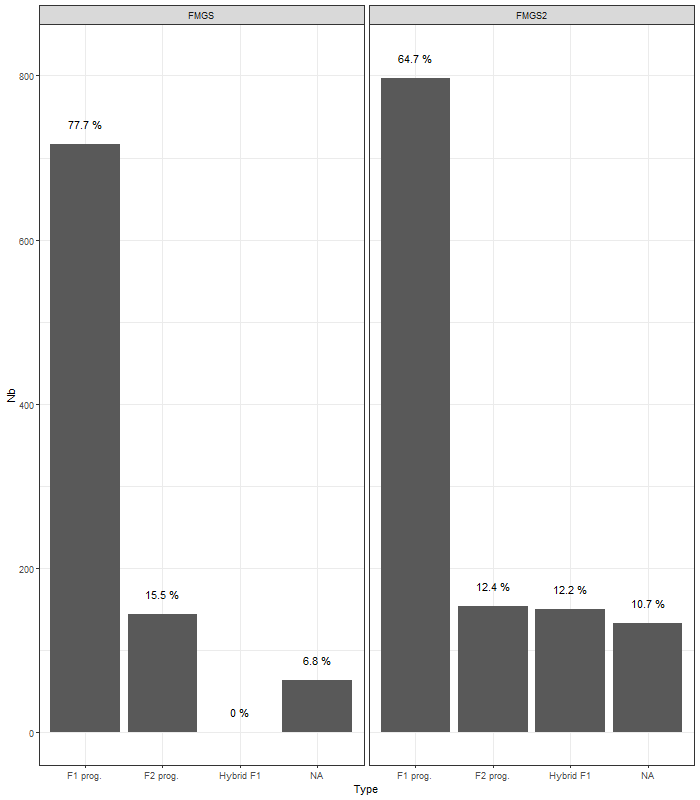

Supplement: Supplementary file 1 [file jof-07-00304-s001.zip › Figure S1.tiff]

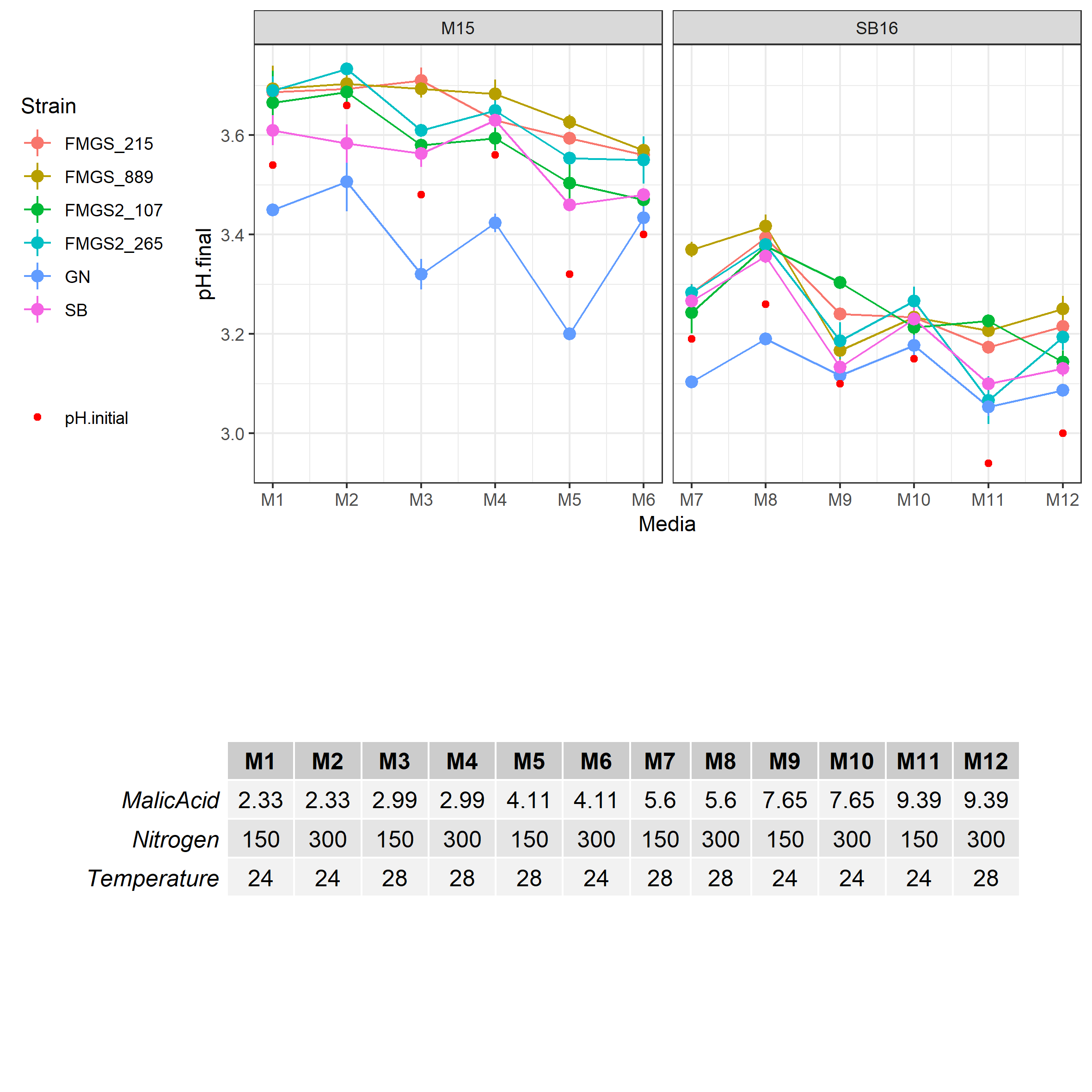

Supplement: Supplementary file 1 [file jof-07-00304-s001.zip › Figure S2.png]

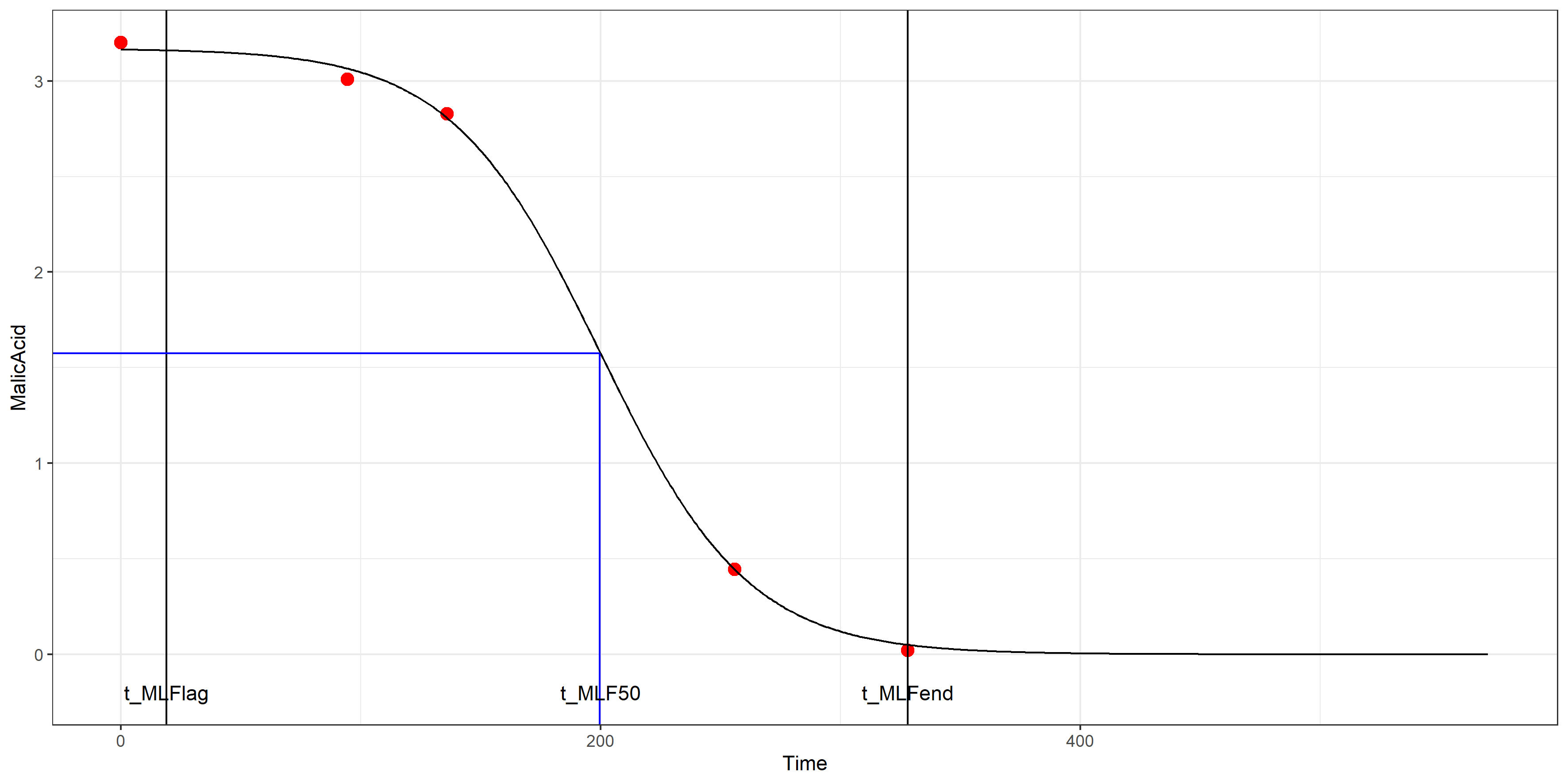

Supplement: Supplementary file 1 [file jof-07-00304-s001.zip › Figure S3.png]
